# Non-destructive prediction of shoot-level leaf area and biomass in *Indocalamus* bamboo via scaling laws

**DOI:** 10.3389/fpls.2025.1650196

**Published:** 2025-09-17

**Authors:** Qinchao Fu, Peijian Shi, Johan Gielis, Karl J. Niklas

**Affiliations:** ^1^ School of Life Science, Leshan Normal University, Leshan, China; ^2^ Bamboo Research Institute, Nanjing Forestry University, Nanjing, China; ^3^ Department of Biosciences Engineering, University of Antwerp, Antwerp, Belgium; ^4^ School of Integrative Plant Science, Cornell University, Ithaca, NY, United States

**Keywords:** Montgomery equation, Montgomery-Koyama-Smith equation, proportional relationship, scaling relationship, total leaf area, total leaf dry mass

## Abstract

This study addresses the critical need for efficient phenotyping methods in plant ecology by exploring predictive models for total leaf area per shoot (*A*_T_) and total leaf dry mass per shoot (*M*_T_), which are both key determinants of photosynthetic capacity and carbon allocation, using two fast-growing bamboo species (*Indocalamus decorus* and *I. longiauritus*) as proof of concept. Traditional approaches to measuring these traits are destructive and labor-intensive, motivating our exploration of non-destructive proxies based on one-dimensional leaf metrics. We validated the Montgomery equation for individual leaves, confirming a robust proportional relationship between leaf area (*A*) and the product of length and width (*LW*) in both *Indocalamus* species (*k* ≈ 0.72). Extending this to the shoot level, the Montgomery-Koyama-Smith equation (MKSE) revealed significant proportionality between total leaf area (*A*_T_) and the composite metric *L*_KS_ *W*_KS_ (where *L*_KS_ denotes the sum of leaf widths and *W*_KS_ denotes maximum leaf length, and the subscript “KS” stands for Koyama-Smith). However, power-law scaling analysis demonstrated allometric, non-isometric relationships for *A*_T_ vs. *L*_KS_ *W*_KS_ (with a scaling exponent α < 1), indicating diminishing leaf area expansion per unit dimensional increase, and *A*_T_ vs. total leaf dry mass (*M*_T_) (α < 1), indicating an increased biomass investment per unit area (i.e., increasing leaf mass per unit area) in larger shoots. These findings validate using simplified one-dimensional metrics that enable accurate, non-destructive predictions of shoot-level functional traits, advancing phenotyping in bamboo ecology, which may hold true more generally for other types of plant species.

## 1 Introduction

Leaves are the primary organ for photosynthetic carbon assimilation in vascular land plants, thereby fundamentally driving ecosystem productivity (Lambers and Poorter, 1992; Wright et al., 2004). Their size, arrangement, and biomass investment are governed by two critical trade-offs. First, a fundamental scaling relationship exists between individual leaf dry mass (*M*) and area (*A*), where larger leaves require disproportionately more structural and hydraulic investments, increasing biomass per unit area (i.e., larger leaf mass per unity area, LMA) and reducing carbon-use efficiency (Shipley, 1995; Milla and Reich, 2007; Niklas et al., 2007; Sack et al., 2012). This reflects a universal trade-off: maximizing photosynthetic area necessitates greater carbon investment per unit area, with LMA serving as a core component of the global leaf economics spectrum (Wright et al., 2004; Poorter et al., 2009). Second, canopy architecture mediates a trade-off between total leaf area per shoot (*A*_T_) and its deployment, as dense foliage packing increases self-shading, significantly reducing photosynthetic efficiency in lower canopy layers (Niklas, 1988; Smith et al., 2017; Wang et al., 2024). Architectural adaptations, such as variations in leaf size, total number (*N*_T_), and phyllotaxy, optimize light capture under this constraint. This optimization is evident in the divergent numerical values for the scaling exponents (α) governing the *A*_T_ vs. *N*_T_ scaling relationship between species with high versus low self-shading canopies (Smith et al., 2017; Koyama and Smith, 2022; Wang et al., 2024).

For example, Smith et al. (2017) report a conserved intraspecific scaling exponent of approximately 0.6 between mean leaf area ( $\bar{A}$ ) and total leaf area per twig (*A*_twig_), reflecting an economic optimization where partitioning larger *A*_twig_ into fewer, larger leaves maximizes carbon gain relative to construction costs under varying self-shading regimes. Similarly, Wang et al. (2024) examined two dwarf bamboo species exhibiting contrasting leaf distributions: *Shibataea chinensis* (with leaves evenly dispersed, and high self-shading) and *Sasaella kongosanensis* ‘Aureostriatus’ (with leaves clustered apically, and low self-shading), and confirmed a power-law scaling relationship between *A*_T_ and *N*_T_ but with divergent scaling exponents: $A_{\text{T}}\propto N_{\text{T}}^{1.128}$ ​ for *S. chinensis* versus $A_{\text{T}}\propto N_{\text{T}}^{0.820}$ ​​ for *S. kongosanensis* ‘Aureostriatus’. This numerical difference in α–values was interpreted to reflect adaptive responses to self-shading, i.e., with pronounced shading in *S. chinensis* driving a disproportionate increase in *A*_T_ with increasing *N*_T_ to compensate for pronounced self-shading, in contrast to minimal shading in *S. kongosanensis* ‘Aureostriatus’ with smaller incremental increases in *A*_T_. In addition, the coefficient of variation in individual leaf area increased with *N*_T_ in both species, highlighting developmental plasticity in leaf size allocation. Collectively, these studies indicate that plant architecture, particularly the integration of branching patterns, phyllotaxy, and leaf trait scaling, mediates a critical trade-off between maximizing photosynthetic area and minimizing the costs imposed by self-shading. The convergence of empirical patterns, such as the conserved $\bar{A}$ vs. *A*_twig_ scaling relationship (Smith et al., 2017), with theoretical optimization models demonstrates that architectural evolution is fundamentally constrained by biophysical economics and hydraulic efficiency (Givnish, 1984; Niklas, 1988; Westoby et al., 2002).

Quantifying the trade-offs between *A*_T_ (which affects light interception) and total leaf dry mass per shoot (*M*_T_, which measures carbon investment) is therefore essential for understanding plant ecological strategies, carbon allocation, and stand-level productivity (Westoby et al., 2002; Poorter et al., 2009). The scaling relationship between *M*_T_ and *A*_T_ also integrates whole-plant economics, dictating photosynthetic efficiency and environmental adaptation (Westoby, 1998; Westoby et al., 2002). Importantly, leaf mass per unit area (LMA) stands as a pivotal metric representing biomass investment per unit light-capturing surface area (Wright et al., 2004). It integrates structural and physiological trade-offs, directly influencing photosynthetic capacity, leaf longevity, and resource use efficiency (Reich et al., 2003; Wright et al., 2004). Leaves characterized by low LMA typically exhibit faster mass-based photosynthetic rates and shorter lifespans, traits associated with resource-rich environments or fast-growing species (Wright et al., 2004; Poorter et al., 2009). Conversely, high LMA indicates a greater investment in structural durability, defense compounds, and longer leaf lifespans, often linked to resource conservation strategies in stressful habitats (Wright et al., 2004; Sterck et al., 2006; Poorter et al., 2009). Consequently, LMA serves as a core component of the global leaf economics spectrum (Wright et al., 2004).

However, although prior work has emphasized the importance of “whole-plant” traits (Westoby et al., 2002), the empirical quantification of the *A*_T_ vs. *M*_T_ scaling relationship across diverse species and environments remains limited. Most studies have focused on interspecific comparisons, which may mask crucial intraspecific variation and plasticity (Poorter et al., 2009) and can be confounded by phylogenetic constraints and differences in plant size or architecture (Westoby, 1998). A significant barrier to addressing these concerns is the destructive and labor-intensive nature of traditional methods for measuring *A*_T_ and *M*_T_, hindering broad studies and large-scale phenotyping (Koyama and Smith, 2022; Wang et al., 2024). Non-destructive proxies offer a solution. For example, building on the Montgomery equation, which assumes that individual leaf area (*A*) is proportional to the product of leaf length (*L*) and width (*W*) (*A* ∝ *LW*) (Montgomery, 1911; Shi et al., 2021), Koyama and Smith (2022) extrapolated this relationship to shoots via the Montgomery-Koyama-Smith equation (MKSE), i.e., $A_{\text{T}}=k_{\text{KS}}L_{\text{KS}}W_{\text{KS}}$ , where *k*_KS_ is a normalization constant, *W*_KS_ is the maximum leaf length per shoot, *L*_KS_ is the sum of leaf widths, which is usually greater than *W*_KS_ for most shoots that have many leaves, and the subscript KS is the acronym for Koyama and Smith (Meng et al., 2025). However, the MKSE assumes that *k*_KS_ is numerically a constant across leaves within any given shoot, an assumption potentially sensitive to architectural heterogeneity, leaf number, and ontogenetic changes during leaf or stem growth, which may limit the accuracy of the MKSE (Meng et al., 2025).

**Figure 1:**
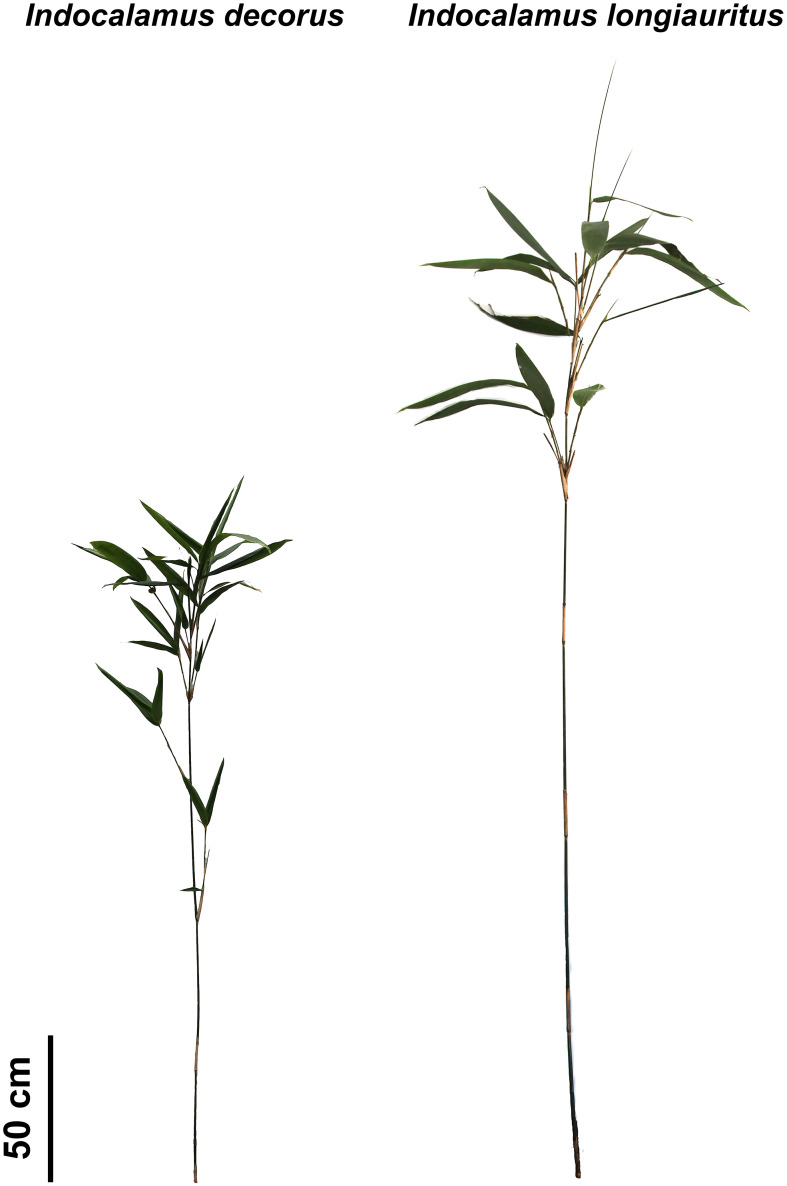
Representative examples of the culms of *I. decorus* (left) and *I. longiauritus* (right).

To explore the utility of the MKSE and the power-law equation (PLE), we examined two species within *Indocalamus*, a genus in the Bambusoideae subfamily (Poaceae), a phylogenetically and ecologically pivotal lineage driving significant carbon sequestration (Vorontsova et al., 2016). The genus *Indocalamus* was selected because it presents an architecturally tractable model due to its large leaves, monopodial rhizomes generating culms bearing a limited number of leaves with pronounced leaf size variation. These features combined with a relatively open canopy architecture enable non-destructive measurements of leaf width, length, and number. We focus on two bamboo species (*I. decorus* and *I. longiauritus*) because of their contrasting leaf-arrangements and internodal lengths (**Figure 1**).

## 2 Materials and methods

### 2.1 Plant material and measurements

A total of 122 *I. decorus* culms were sampled in Hongya County, Meishan, Sichuan Province, China (103°27’53’’ E, 29°55’33’’ N, 504.5 m a.s.l.), and a total of 120 *I. longiauritus* culms were sampled on the Leshan Normal University Campus, Leshan, Sichuan Province, China (103°44’57’’ E, 29°33’54’’ N, 419.6 m a.s.l.) in November 2024. *Indocalamus decorus* is native to Meishan, whereas *I. longiauritus* was introduced from the White Horse Experimental Station of Nanjing Forestry University, Jiangsu Province, China to the current sampling site in 2018, where it has naturalized on the campus. All sampled shoots originated from sprouts appearing in spring 2023. Sampling occurred near the end of their second growth season, ensuring that shoots were mature and leaves had fully matured. Sampling sites for *I. decorus* and *I. longiauritus* were selected to ensure minimal anthropogenic disturbance and climatic consistency and similarity. Both locations represent naturalized growth: *I. decorus* in its native habitat and *I. longiauritus* naturalized since 2018 without subsequent management. The close distance of 48.6 km between sites minimizes climatic differences, while architectural simplicity (low leaf counts and open canopies) facilitated accurate non-destructive trait measurements. Sampling at this time of year ensured full leaf maturation and dry mass stabilization (i.e., completion of leaf expansion (lamina size stabilized) and sclerification, eliminating ontogenetic biases in area/mass relationships). This rendered oven-drying protocols following standardized methods sufficient to achieve constant dry weight, ensuring stable biomass measurements.

The lamina length (*L*) and width (*W*) of each leaf of the 242 shoots were measured. According to prior studies (Shi et al., 2021), individual leaf lamina area (*A*) can be estimated as the product of *L* and *W* multiplied by a proportionality coefficient (*k*, called the Montgomery parameter) called the Montgomery equation, i.e., *A* = *kLW*) as proposed by Montgomery (1911). To determine the numerical value of *k*, 254 *I. decorus* leaves and 251 *I. longiauritus* leaves were scanned using a photo scanner (M208, BenQ Corporation, Shanghai, China). The leaf images were transferred to black-and-white images in bitmap (bmp) format, and the Matlab procedure proposed by Su et al. (2019) was used to extract the planar coordinates for each lamina. The “bilat’ function in the R packaged “biogeom” (v.1.4.3) based on R (v4.3.1; R Core Team, 2023) was used to determine *A*, *L*, and *W* for each leaf. For each shoot, all the leaves were subsequently dried in an oven (DHG-9240A, Shanghai Yiheng Scientific Instruments Co., Ltd., Shanghai, China) at 105°C for 30 min and then continuously at 75°C until achieving a constant dry weight. The dried leaves for each shoot were measured to determine dry mass using an electronic balance (ML203; Mettler Toledo Company, Greifensee, Switzerland; measurement accuracy 0.001 g).

Total leaf area per shoot (*A*_T_) was obtained by summing the individual leaf areas per shoot; the sum of individual leaf widths on a shoot is referred to as *W*_KS_; and the maximum individual leaf length on a shoot is referred to as *L*_KS._ The subscript KS is used in honor of Koyama and Smith.

The raw data of the length and width for all leaves on the 242 shoots, those of the length, width and area for the 505 randomly sampled leaves (used to estimate the Montgomery parameter), and those of the total leaf dry mass for each of the 242 shoots of the two bamboo species are assessable from **Supplementary Tables S1**−**S3**, respectively.

### 2.2 Statistical analysis

The frequency distributions of the number of leaves, total leaf mass, and total leaf area per shoot of the two bamboo species were modeled using the normal, log-normal, and two-parameter Weibull probability density functions. The Weibull probability density function takes the form (Weibull, 1951):


(1) $$f\left(x\right)=\frac{\delta }{\lambda }{\left(\frac{x}{\lambda }\right)}^{\delta -1}\text{exp}\left[-{\left(\frac{x}{\lambda }\right)}^{\delta }\right]$$

where *δ* is the shape parameter, and *λ* is the scale parameter. The two parameters in Equation 1 were estimated using maximum likelihood estimation (MLE) which was carried out by the “mle2” function in the R package bbmle (v1.0.25.1). The numerical value of the parameter *δ* can be used to determine whether the distribution is left-skewed, right-skewed or symmetrical, i.e., *δ* < 3.6 indicates a right-skewed distribution; *δ* > 3.6 indicates a left-skewed distribution is indicated; and *δ* = 3.6 indicates a symmetrical distribution (Murthy et al., 2004).

The log-transformed version of the Montgomery equation (Montgomery, 1911; Shi et al., 2021) was fitted to determine the Montgomery parameter:


(2) log(A)=log(k)+log(LW)

We tested the validity of the Koyama and Smith (2022) model, which assumes a one-to-one proportional relationship between *A*_T_ and *L*_KS_ *W*_KS_ (which is referred to as the Montgomery-Koyama-Smith equation, denoted as MKSE), i.e.,


(3) $$A_{\text{T}}=k_{\text{KS}}L_{\text{KS}}W_{\text{KS}}$$

where *k*_KS_ is the proportionality coefficient of the MKSE. To stabilize the variances of *A*_T_ and *L*_KS_ *W*_KS_, we also used the log-transform of Equation 3 for carrying out a linear fitting:


(4) $$\log\left(A_{\text{T}}\right)=\log\left(k_{\text{KS}}\right)+\log\left(L_{\text{KS}}W_{\text{KS}}\right)$$

Across diverse plant species, two interdependent variables of *Y*_1_ and *Y*_2_, such as leaf area and mass, are frequently found to follow a power-law equation (denoted as PLE) (Niklas, 1994):


(5) $$Y_{2}=\text{β}Y_{1}^{\text{α}}$$

where β is the normalization constant and α is the scaling exponent (i.e., the rate of change in *Y*_2_ with respect to *Y*_1_; Niklas, 1994) Because $\text{α}=\frac{dY_{2}/Y_{2}}{dY_{1}/Y_{1}}$ , it follows that (i) α > 1 indicates that increases in *Y*_1_ do not keep pace with the increases in *Y*_2_; (ii) α < 1 indicates that increases in *Y*_2_ do not keep pace with the increases in *Y*_1_; and (iii) α = 1 indicates an isometric relationship between *Y*_1_ and *Y*_2_. Cases (i) and (ii) are referred to as allometric scaling relationships, whereas case (iii) is referred to as an isometric scaling relationship. We examined whether the *A*_T_ vs. *L*_KS_ *W*_KS_ relationship tends to be allometric or isometric. Log-transformation of Equation 5 takes the form:


(6) y=γ+αx

where *y* = log(*Y*_2_), *x* = log(*Y*_1_), and γ = log(β) is the log-log *y*-intercept. Equation (5) was used to describe the probable scaling relationship between *A*_T_ and *L*_KS_ *W*_KS_. In addition, it was also used to describe the scaling relationships between *A*_T_ and the total number of leaves per shoot (*N*_T_), and between *A*_T_ and *M*_T_, where *M*_T_ represents total leaf dry mass per shoot.

Given that *LW* has the same physical dimensions as *A*, and *L*_KS_ *W*_KS_ has the same dimensions as *A*_T_, common least-squares regression was used to fit Equations 2, 4 and 6. However, both *A*_T_ and *N*_T_, and *A*_T_ and *M*_T_ have different physical dimensions. Thus, reduced major axis protocols (Niklas, 1994; Quinn and Keough, 2002) were used to estimate the slope and *y*-intercept of the *A*_T_ vs. *N*_T_ and the *A*_T_ vs. *M*_T_ scaling relationships. The root-mean-square error (RMSE) and coefficient of determination (*r* ^2^) were used to assess the goodness of fit of the linear fitting.

The bootstrap percentile method (Efron and Tibshirani, 1993) was used to calculate the 95% confidence intervals (CIs) of α and β. The bootstrap percentile method based on 3000 bootstrap replicates was used to test the significance of the difference between any two scaling exponents and any two normalization constants between the two bamboo species. If the 95% CI of the differences between the bootstrap replicates of one scaling exponent (or one normalization constant) and those of another scaling exponent (or another normalization constant) included zero, the difference between the two statistical parameters was judged not to be significant; if the 95% CI did not include zero, the difference was judged to be statistically significant.

All analyses were conducted in R (v4.3.1; R Core Team, 2023).

## 3 Results

**Figure 2:**
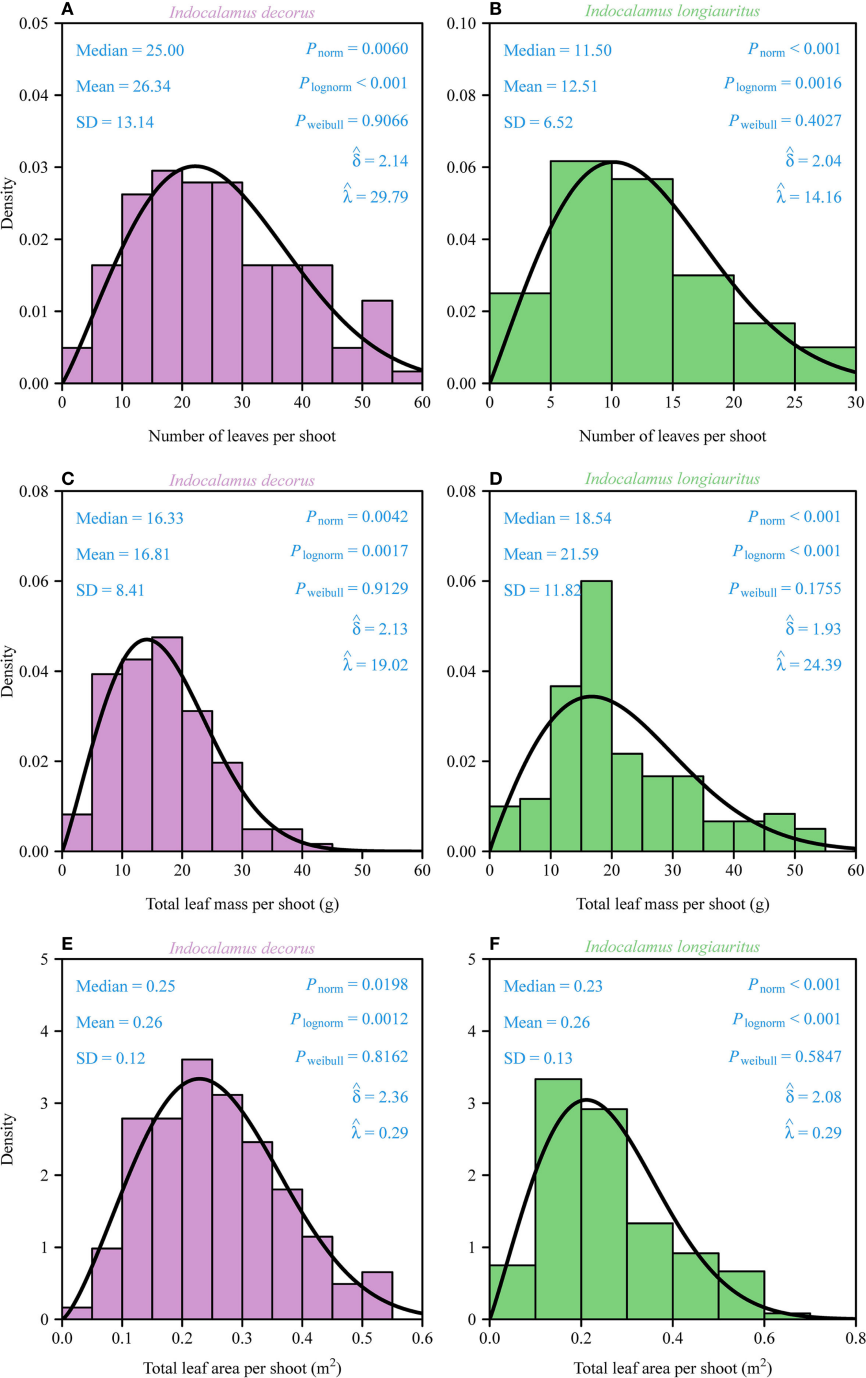
Density distributions of the total number of leaves, total leaf dry mass, and total leaf area per shoot for *I. decorus* **(A, C, E)** and *I. longiauritus* **(B, D, F)**. SD is the standard error; *P*_norm_ is the probability that the data are consistent with the null hypothesis of a normal distribution; *P*_lognorm_ is the probability that the data are consistent with the null hypothesis of a log-normal distribution; *P*_weibull_ is the probability that the data are consistent with the null hypothesis of a Weibull distribution. $\hat{\delta }$ and $\hat{\lambda }$ are the estimates of the shape and scale parameters in the Weibull probability density function. The solid curves represent the predicted Weibull probability densities.

The distributions of the number of leaves, total leaf mass, and total leaf area per shoot in both bamboo species were assessed using normal, log-normal, and two-parameter Weibull probability density functions (Section 2.2). Kolmogorov-Smirnov goodness-of-fit tests revealed that the data for all three traits in both species were best described by right-skewed Weibull distributions (all *P*_Weibull_ > 0.05, *δ <* 3.6; **Figure 2**). In contrast, the normal and log-normal distributions provided significantly poorer fits (all *P*_norm_< 0.05 and *P*_lognorm_< 0.05, respectively; **Figure 2**), justifying the selection of the Weibull model. Despite taxonomic congruence at the genus level, *I. decorus* exhibited a significantly higher mean leaf count per shoot (26.34 ± 13.14, mean ± SD) compared to *I. longiauritus* (12.51 ± 6.52). The twofold difference between the two species was interpreted to reflect interspecies variation in foliar architecture. However, there was a negligible difference in total leaf area between the two species (**Figures 2E, F**).

**Figure 3:**
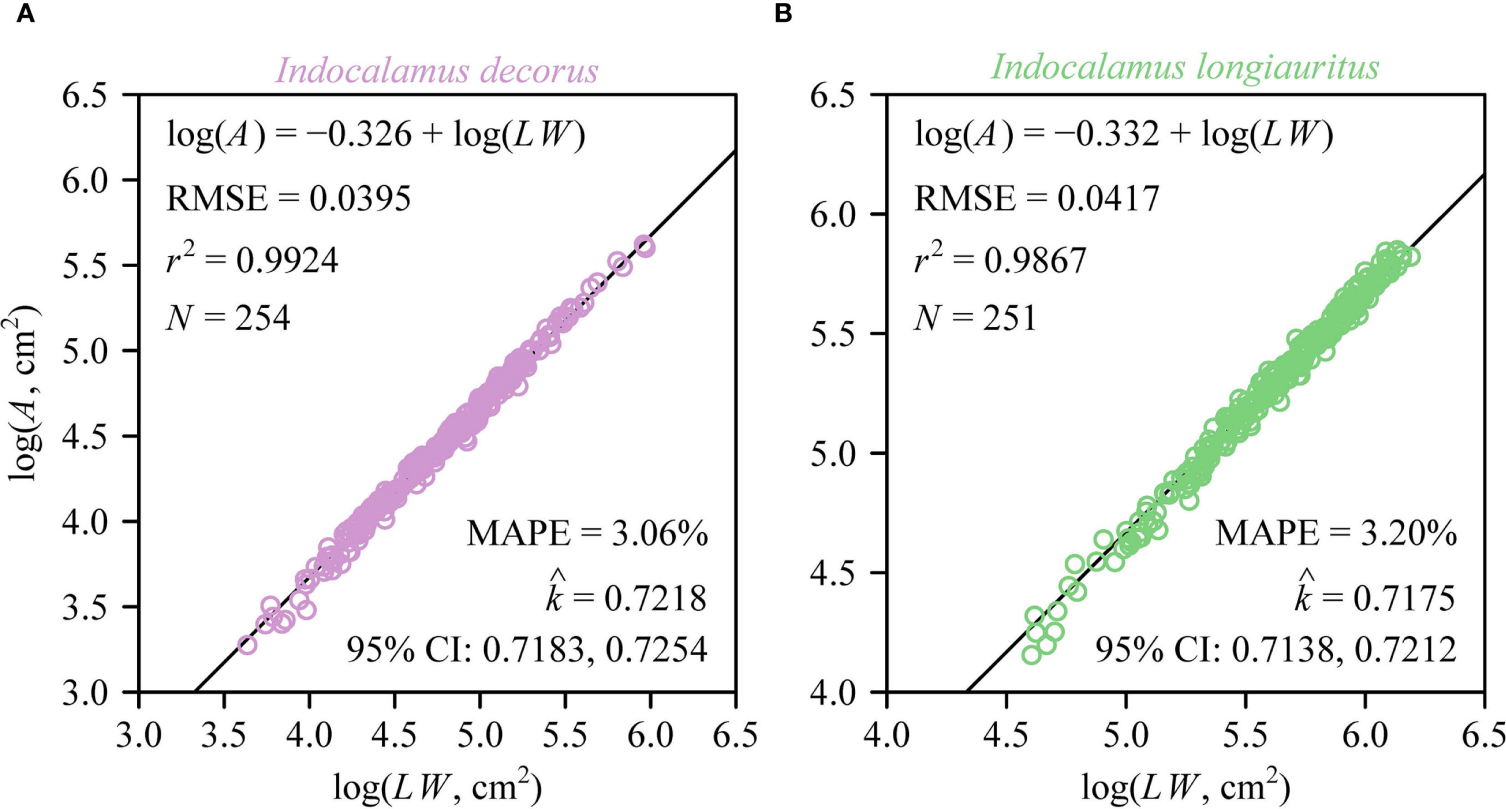
Fitted log-log bivariate plots of the relationships between leaf area *(A)* and the product of leaf length and leaf width (*LW*) for *I. decorus* **(A)** and *I. longiauritus* **(B)**. RMSE is the root-mean-square error of the linear fit; *r* ^2^ is the coefficient of determination; *N* is the sample size; MAPE is the mean absolute percent error between the observed and predicted leaf areas; $\hat{k}$ is the estimated Montgomery parameter, i.e., the estimated proportionality coefficient between *A* and *LW*; 95% CI represents the 95% confidence interval of the estimated Montgomery parameter.

**Figure 4:**
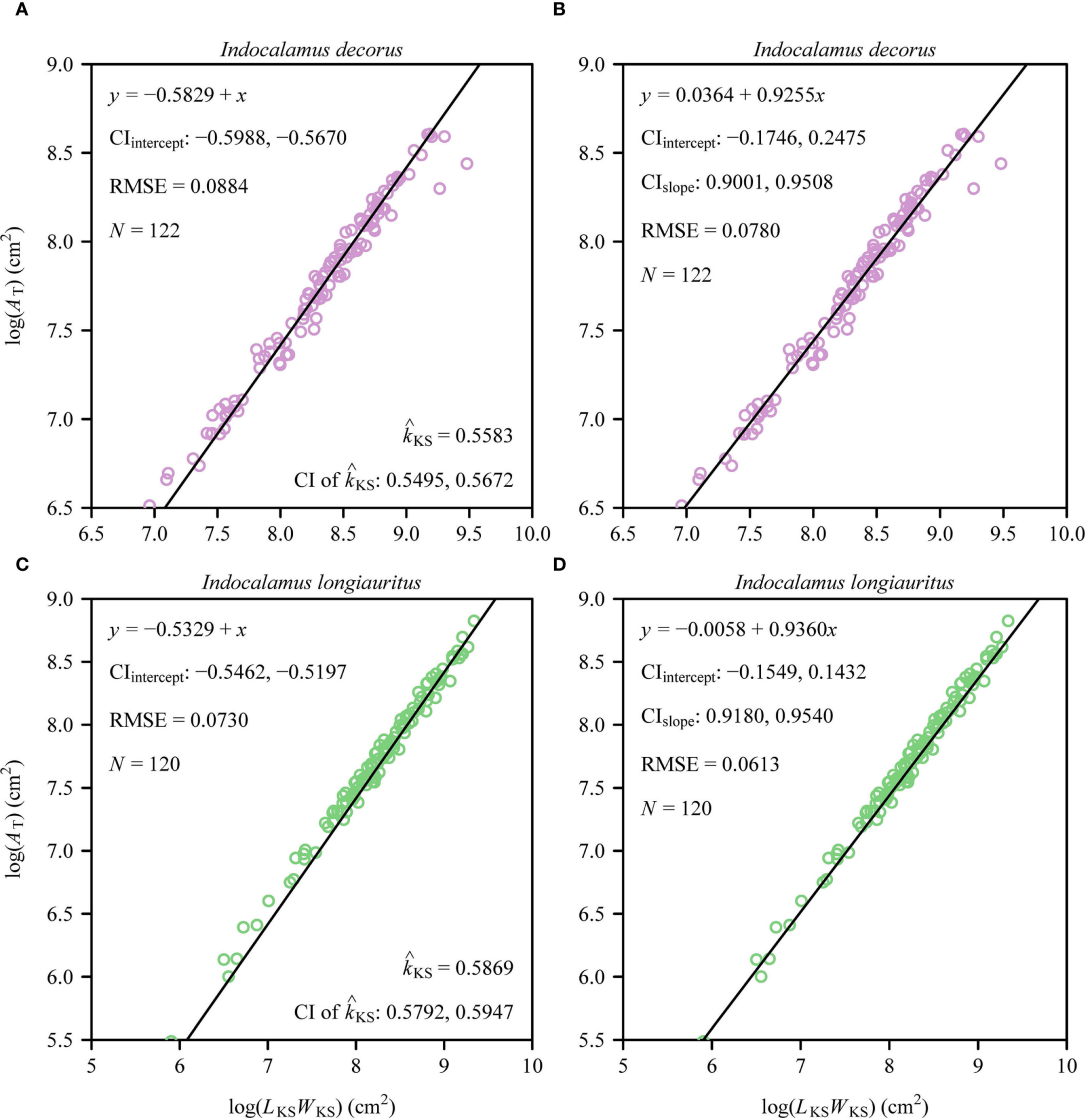
Fitted log-log bivariate plots of the relationships between total leaf area per shoot (*A*_T_) and the product of the sum of individual leaf widths (*L*_KS_) and the maximum individual leaf length (*W*_KS_) for *I. decorus* **(A, B)** and *I. longiauritus* **(C, D)**. Panels **(A, C)** show the fitted results using the Montgomery-Koyama-Smith equation that hypothesizes the log-log slope to be unity, and panels **(B, D)** show the fitted results using the power-law equation. The open circles represent the observations; the solids represent the regression lines; CI_intercept_ represents the 95% confidence interval of the estimated *y*-intercept; CI_slope_ represents the 95% confidence interval of the estimated slope; ${\hat{k}}_{\text{KS}}$ is the estimated proportionality coefficient of the MKSE; CI of ${\hat{k}}_{\text{KS}}$ represents the 95% confidence interval of the estimated proportionality coefficient of the MKSE; RMSE is the root-mean-square error of the linear fitting; and *N* is the sample size, i.e., the number of shoots.

**Figure 5:**
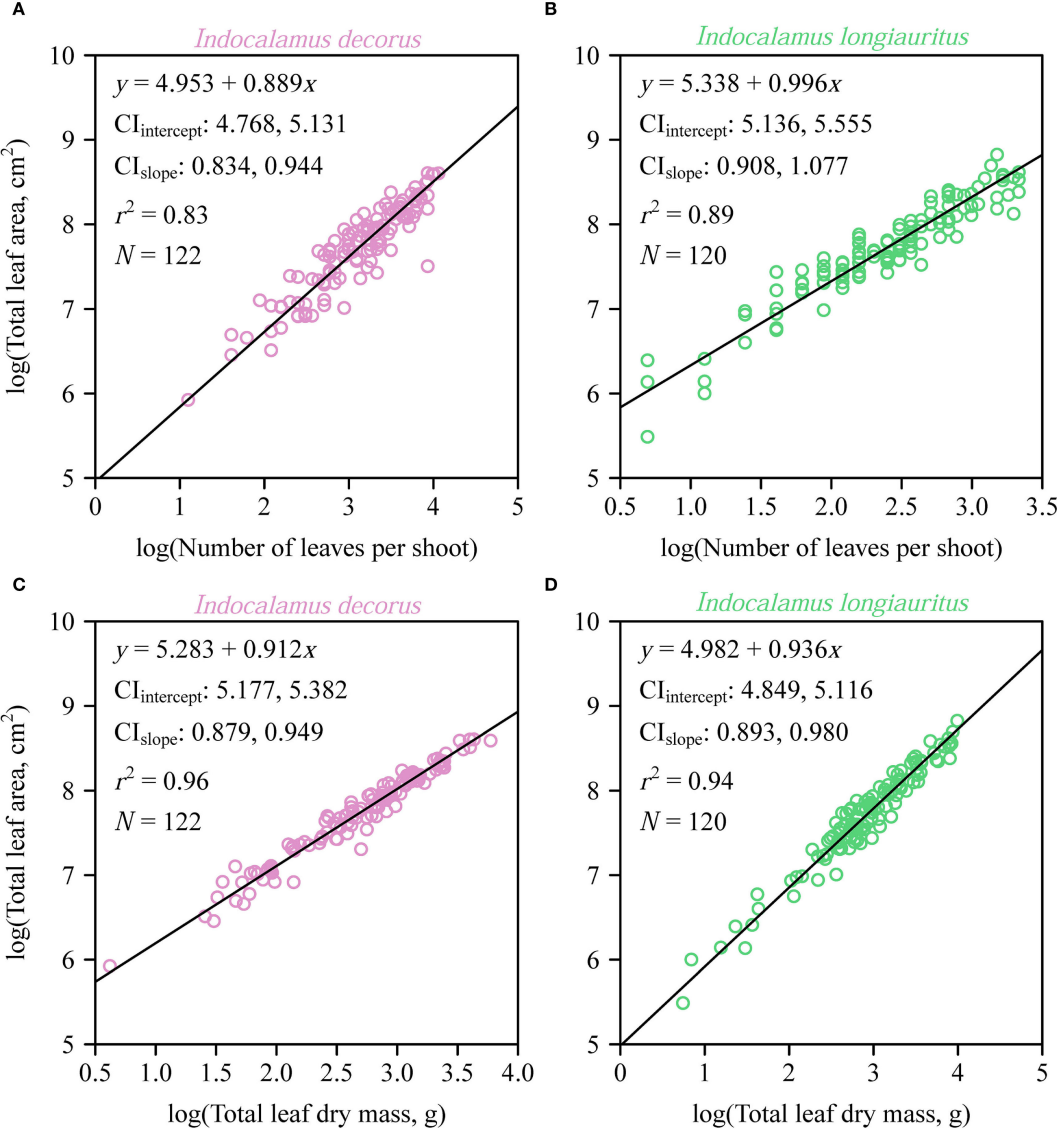
Fitted log-log bivariate plots of the relationships between total leaf area per shoot (*A*_T_) and the number of leaves per shoot, and between *A*_T_ and the total leaf dry mass for *Indocalamus decorus* **(A, C)** and *I. longiauritus* **(B, D)**. The open circles represent the observations; the solids represent the regression lines; CI_intercept_ represents the 95% confidence interval of the estimated *y*-intercept; CI_slope_ represents the 95% confidence interval of the estimated slope; *r* ^2^ is the coefficient of determination; and *N* is the sample size, i.e., the number of shoots.

Individual leaf area (*A*) exhibited a strong proportional relationship with the product of leaf length and width (*LW*) in both species as predicted by the Montgomery equation (**Figure 3**). The estimated Montgomery parameter (*k*) was the same between the two species ( ≈ 0.72), with high coefficients of determination (> 0.98) and low prediction errors (RMSE < 0.05). At the shoot level, a significant proportionality was observed between total leaf area (*A*_T_) and the product of the sum of leaf widths (*L*_KS_) and maximum leaf length (*W*_KS_), as described by the MKSE (**Figures 4A, C**). The proportionality coefficients (*k*_KS_) of the two species were not statistically significantly different. Regression analyses confirmed allometric rather than isometric scaling relationships for *A*_T_ vs. *L*_KS_ *W*_KS_ (α < 1; **Figures 4B, D**), indicating diminishing area gains per unit increase in the dimensional composite, and for *A*_T_ and *M*_T_ (α < 1; **Figure 5**), revealing increasing biomass investment per unit leaf area in larger shoots. Bootstrap analyses confirmed that the scaling exponents differed significantly from isometry (95% CIs excluded unity) for both relationships. There was a significant difference in the estimated scaling exponents of *A*_T_ vs. *N*_T_ between the two species, i.e., the upper bound of the 95% CI was smaller than unity for *I. decorus*, whereas the 95% CI of *A*_T_ vs. *N*_T_ included unity for *I. longiauritus*.

## 4 Discussion

The development of non-destructive methods to quantify functional traits such as total leaf area per shoot (*A*_T_) and total leaf dry mass per shoot (*M*_T_) represents an important advance in plant ecology, particularly for economically and ecologically significant taxa such as *Indocalamus* bamboos. Our study provides promising predictive models for the measurement of these traits using simplified one-dimensional leaf metrics, which is shown to be valid for two representative and important bamboo species (*I. decorus* and *I. longiauritus*). The three key findings of this study are the identification of (1) a strong proportionality for individual leaves via the Montgomery equation (*A* ∝ *LW*), (2) a species-specific proportionality at the shoot level using the Montgomery-Koyama-Smith equation (MKSE, *A*_T_ ∝ *L*_KS_ *W*_KS_), and (3) allometric *A*_T_ vs. *L*_KS_ *W*_KS_, *A*_T_ vs. *N*_T_ and *A*_T_ vs. *M*_T_ scaling relationships, which reveal fundamental architectural and allocational constraints. These findings are contextualized in the following sections through three interrelated lenses: (1) the mechanistic basis of the *A*_T_ vs. *L*_KS_ *W*_KS_ allometry, (2) the ecological drivers of divergent *A*_T_ vs. *N*_T_ scaling relationships between species, and (3) the implications of the *A*_T_ vs. *M*_T_ allometry for carbon investment strategies.

### 4.1 *A*_T_ vs. *L*_KS_ *W*_KS_ allometry: architectural constraints and the limits of proportionality

The MKSE posits a strict isometric scaling relationship (α = 1) between *A*_T_ and the composite metric *L*_KS_ *W*_KS_ (i.e., the product of the sum of leaf widths and maximum leaf length). The rationale for assuming constant *k*_KS_ in the MKSE merges from the foundational Montgomery equation (individual leaf area *A* ∝ *LW*), where a species-specific constant *k* captures the geometric proportionality between *A* and the *LW* product. Extending this proportionality to the shoot level (*A*_T_ ∝ *L*_KS_ *W*_KS_) implicitly assumes that the average proportionality (*k*) across all leaves within a shoot remains constant, or that variations cancel out, allowing *L*_KS_ *W*_KS_ to serve as a dimensionally consistent proxy for total area. Furthermore, prior theoretical work (Meng et al., 2025) shows that, if four strict conditions hold true simultaneously for shoots, *A*_T_ scales isometrically with *L*_KS_ *W*_KS_ (α = 1): (1) the number of leaves is constant across shoots, (2) individual leaf areas sorted in ascending order for each shoot follow a geometric series, (3) the common ratio of this geometric series is constant across shoots, and (4) individual leaf width scales (isometrically or allometrically) with leaf length. However, our analyses reveal a significant sub-linearity (α < 1) in both species (**Figures 4B, D**), indicating that *A*_T_ increases at a disproportionately slower rate than *L*_KS_ *W*_KS_ as shoots increase in overall size. This deviation from the idealized MKSE proportionality arises from intrinsic architectural heterogeneities neglected in the model’s assumptions. The MKSE requires that the numerical value of the Montgomery parameter (*k*) is constant or nearly so across all leaves per shoot (Koyama and Smith, 2022). However, in the case of *Indocalamus*, pronounced intra-shoot leaf size variation is evident, which is interpreted to reflect an ontogenetic gradient in leaf development along the length of shoots (e.g., smaller basal leaves vs. larger apical leaves), which results in a basipetal shift in the numerical value of *k* (Shi et al., 2021). As shoot size increases, larger leaves tend to exhibit lower *k*-values due to the thickening of vascular bundles and the mesophyll, reducing the area gained per unit *LW* (Meng et al., 2025). Consequently, *L*_KS_ *W*_KS_ overestimates *A*_T_ in larger shoots, as dimensional increases in *L*_KS_ *W*_KS_ outpace actual area expansion (*A*_T_). This aligns with the results reported by Meng et al. (2025), who observed that MKSE yields less than optimal results using *Sasaella kongosanensis* ‘Aureostriatus’, presumably because of inconsistent leaf-number-dependent allometries. In the case of *Indocalamus*, architectural simplicity (low leaf counts, and open canopies) likely reduces (but does not eliminate) this bias, highlighting the fact that plants tend to optimize leaf deployment not for geometric proportionality but for light capture efficiency as a consequence of hydraulic and mechanical trade-offs (Givnish, 1984; Niklas, 1994; Westoby et al., 2002). Thus, scaling exponents with numerical values less than unity (α < 1) identify the “cost” of producing larger shoots, where diminishing returns in area gain per unit dimensional investment reflect selection for reducing self-shading and enhancing hydraulic safety (Smith et al., 2017; Wang et al., 2024).

Our findings confirm that structural heterogeneity within shoots, particularly the ontogenetic gradient in leaf size and associated variation in the Montgomery parameter (*k*), is a key driver of the observed deviation from strict proportionality in the MKSE. It is therefore acknowledged that intra-shoot variability in *k*-values represents a potential source of prediction error, especially in shoots with more complex architecture or pronounced developmental gradients. Future studies should focus on the quantification of *k*-value variation across different leaf sizes, phyllotaxies, and developmental stages. Such analyses are crucial for assessing the robustness of the MKSE across diverse plant forms and for developing refined predictive models that account for inherent structural heterogeneity.

The nonlinear scaling of *A*_T_ vs. *L*_KS_ *W*_KS_ observed when α < 1 also mirrors light attenuation patterns in tropical canopies. Kitajima et al. (2005) quantified how late-successional trees with orthotropic branching enhance shading through high cumulative leaf area index (LAI) ranging between 5 and 8 despite low light extinction coefficients (*K* = 0.35), whereas pioneers maintain minimal LAI (< 0.5) for maximizing light penetration. In the case of *Indocalamus*, similar architectural optimization occurs, e.g., larger shoots exhibit diminishing area gains per unit dimensional increases (*L*_KS_ *W*_KS_), reflecting trade-offs between hydraulic safety and light capture efficiency. Although our models demonstrate robustness in the studied populations, environmental factors (e.g., light, water, or nutrient gradients) may influence scaling parameters in heterogeneous habitats, e.g., resource limitation could exacerbate diminishing returns in the scaling of *A*_T_/*L*_KS_ *W*_KS_ (α < 1) by constraining leaf expansion. Future studies manipulating environmental stressors are needed to test the plasticity of these relationships across ecological settings.

### 4.2 Divergent *A*_T_ vs. *N*_T_ scaling relationships: self-shading, canopy architecture, and species-specific strategies

Our data reveal an interspecific contrast in how *A*_T_ scales with respect to leaf number (*N*_T_) in two closely related species. Specifically, we observe an allometric *A_T_* vs. *N_T_* scaling relationship in the case of *I. decorus* (α < 1) and an isometric scaling relationship in the case of *I. longiauritus* (α ≈ 1) (**Figures 5A, B**). We interpret this difference to reflect adaptations to divergent self-shading intensity, modulated by canopy architecture. Specifically, *I. decorus* shoots, on average, bear twice as many leaves per shoot (mean *N*_T_ = 26.3) compared to *I. longiauritus* (mean *N*_T_ ​= 12.5), and consequently experience significantly more self-shading. Consequently, *I. decorus* exhibits an allometric scaling relationship (i.e., $A_{\text{T}}\propto N_{\text{T}}^{0.889}$ ), such that the addition of each leaf contributes disproportionately more to total leaf area per shoot. This observation differs from the data reported by Wang et al. (2024), who observed an *A*_T_ vs. *N*_T_ scaling relationship governed by α = 1.128 in *Shibataea chinensis* (experiencing considerable self-shading), compared to α = 0.820 in *Sasaella kongosanensis* ‘Aureostriatus’ (experiencing little or no self-shading). The difference between α > 1 in *S. chinensis* and α < 1 in *I. decorus* (both high self-shading species) likely reflects distinct architectural adaptations to shading. *S. chinensis* mitigates shading by means of an even vertical leaf distribution, where the addition of leaves necessitates disproportionate area increases (α > 1) to compensate for layered shading. In contrast, *I. decorus* exhibits apical leaf clustering, where adding leaves expands existing clusters rather than adding new shaded layers. This architecture imposes stronger self-thinning constraints, limiting area gains per added leaf (α < 1) despite high overall shading. Thus, though both species experience considerable self-shading, their divergent leaf deployment strategies drive contrasting scaling exponents. Thus, the numerical value of the *A*_T_ vs. *N*_T_ scaling exponent emerges as an important functional signature of species-specific light-harvesting optimization as summarized by Corner’s rules (Corner, 1949), which proposes that branching geometry dictates whether area expansion prioritizes leaf size or number (Olson et al., 2018).

Our observations of crown architecture also align with recent discoveries of life-form-dependent shade avoidance strategies. For example, Aoyagi et al. (2024) have shown that Japanese monoaxial trees exhibit divergent leaf arrangements, i.e., deciduous species minimize self-shading through top-down (basipetal) decreasing petiole lengths (*r* = −0.94) and deflection angles, enabling apical leaf expansion, whereas evergreen species employ bottom-up (acropetal) increasing angles (*r* = 0.82) to create vertical inter-whorl spacing. This dichotomy underscores how phylogenetic constraints shape crown plasticity, which is evident in *I. decorus* (high leaf count) and *I. longiauritus* (low leaf count), where architectural simplicity permits flexible adaptations to self-shading.

### 4.3 *A*_T_ vs. *M*_T_ allometry and its implications for biomass investment efficiency

We suspect that the *A_T_* vs. *M_T_* allometry integrates two key trade-offs: (i) potentially competing leaf-level structural and physiological requirements, and (ii) shoot-level leaf biomass partitioning trade-offs. Across diverse species, individual leaves tend to exhibit a positive *M* vs. *A* scaling allometry ( $M\propto A^{\alpha >1}$ ), wherein larger leaves require disproportionately more mass with increasing lamina area, presumably for mechanical and hydraulic tissue systems (Milla and Reich, 2007; Niklas et al., 2007; Niinemets et al., 2007). However, in the case of *Indocalamus*, larger shoots appear to produce thinner leaves or leaves with reduced tissue density to optimize carbon allocation. In *Indocalamus*, both mechanisms likely operate. First, the conserved Montgomery parameter (*k* ≈ 0.72) indicates geometric similarity in leaf shape, but the shoot-level *A*_T_ vs. *M*_T_ allometry (α = 0.912 and 0.936 for the two investigated bamboo species) indicates increasing mass per unit area. This agrees with the “diminishing returns” pattern observed in subtropical ferns, where total leaf area scales allometrically with biomass (α < 1.0) across elevations (Chen et al., 2023). This further indicates that there are similar resource allocation strategies governing the *A*_T_ vs. *M*_T_ scaling relationship, i.e., increased individual plant (or shoot) size requires increases in LMA to enhance structural support under variable light regimes. Second, as shoots enlarge, resource allocation shifts toward higher LMA. This increased investment per unit area supports greater structural integrity (e.g., thicker mesophyll, denser vascular bundles) and potentially enhanced longevity or defense, which may be advantageous in environments where larger shoots experience greater mechanical stress or herbivory pressure, even within generally resource-rich habitats (Wright et al., 2004; Poorter et al., 2009). Importantly, the *A*_T_ vs. *M*_T_ allometry (α < 1) confirms the “diminishing returns” pattern reported for whole-plant LMA scaling (Niklas et al., 2007). This reflects an economic strategy where larger shoots exhibit a relative increase in biomass investment per unit photosynthetic area (higher LMA). While this prioritization of structural biomass over maximal area expansion (*A*_T_/*M*_T_ decreases as *M*_T_ increases) might seem to constrain potential photosynthetic gains per unit carbon invested, it likely enhances hydraulic safety, mechanical stability, and leaf longevity in larger architectural units. This strategy optimizes carbon gain under the specific constraints faced by larger shoots (e.g., greater self-loading, longer hydraulic pathways), rather than maximizing area per se under non-limiting conditions (Poorter et al., 2009). The conserved low average LMA characteristic of fast-growing bamboos like *Indocalamus* (Wright et al., 2004) is thus achieved alongside this ontogenetic increase in LMA within shoots as they develop.

## 5 Conclusions

This study fulfills the critical need for efficient, non-destructive phenotyping methods in plant ecology by establishing robust predictive models for total leaf area per shoot (*A*_T_) and total leaf dry mass per shoot (*M*_T_), as shown for two *Indocalamus* bamboo species. The validation of the Montgomery equation confirms a strong proportional relationship between individual leaf area (*A*) and the product of leaf length and width (*LW*) for both *I. decorus* and *I. longiauritus*, with statistically equivalent Montgomery parameters (*k* ≈ 0.72) and high predictive accuracy across the two species. Extending this principle to the shoot level, the data show that the Montgomery-Koyama-Smith equation (MKSE) captures the proportionality between *A*_T_ and the composite metric *L*_KS_ *W*_KS_ (the product of the sum of leaf widths and maximum leaf length), although species-specific coefficients highlight nuanced biological variation. Crucially, power-law scaling analyses confirm pervasive allometric relationships, i.e., the scaling exponent (α < 1) for *A*_T_ versus *L*_KS_ *W*_KS_ indicates that leaf area expansion lags behind dimensional increases, reflecting architectural constraints on light-harvesting efficiency. Similarly, the allometric scaling (α < 1) between *A*_T_ and *M*_T_ reveals increasing biomass investment per unit leaf area in larger shoots, indicating an increase in leaf mass per unit area (LMA) with shoot development. This non-isometric investment strategy optimizes carbon allocation by prioritizing structural biomass over photosynthetic surface expansion as shoots grow, aligning with ecological optimizations for hydraulic safety and durability under increasing size constraints. Methodologically, our findings validate that simplified one-dimensional leaf metrics can be used to accurately predict shoot-level functional traits, bypassing labor-intensive destructive sampling. The architectural simplicity of *Indocalamus*, which is characterized by limited leaf counts, pronounced size variation, and open canopies, proved instrumental in minimizing noise from branching complexity, thereby enhancing model robustness. However, the observed sensitivity of proportionality constants to species identity and shoot size indicates the need for taxon-specific calibrations when applying the MKSE across diverse bamboo lineages or complex plant architectures. Future research should explore (1) the transferability of these models to other genera, (2) the effects of environmental modulators of scaling exponents, and (3) integrate remote-sensing technologies for field-scale phenotyping. As Valladares and Niinemets (2008) have shown, elevated CO_2_ may temporarily enhance carbon gain in shade-tolerant species, but concurrent warming and fragmentation threaten their understory niches, especially given low phenotypic plasticity. Our models for *Indocalamus* suggest that allometric *A*_T_ vs. *M*_T_ scaling (α < 1) could mitigate these pressures by prioritizing photosynthetic area over structural biomass, offering a buffer against environmental instability. Research focused on projected climate effects on shade-adapted species should account for multifactorial stressors.

## Data Availability

The original contributions presented in the study are included in the article/**Supplementary Material**. Further inquiries can be directed to the corresponding authors.
